# NPC-26 kills human colorectal cancer cells via activating AMPK signaling

**DOI:** 10.18632/oncotarget.15436

**Published:** 2017-02-17

**Authors:** Zhen Zhao, Li Feng, Jiqin Wang, Deshan Cheng, Mei Liu, Meirong Ling, Weiping Xu, Keyu Sun

**Affiliations:** ^1^ Clinical Laboratory, Minhang Hospital, Fudan University, Shanghai, China; ^2^ Department of Gastroenterology, Minhang Hospital, Fudan University, Shanghai, China; ^3^ Emergency Department, Minhang Hospital, Fudan University, Shanghai, China; ^4^ Shanghai University of Medicine & Health Sciences, Shanghai, China

**Keywords:** NPC-26, colorectal cancer, AMP-activated protein kinase (AMPK), mitochondrion, cell death

## Abstract

NPC-26 is novel mitochondrion-interfering compound. The current study tested its potential effect against colorectal cancer (CRC) cells. We demonstrated that NPC-26 induced potent anti-proliferative and cytotoxic activities against CRC cell lines (HCT-116, DLD-1 and HT-29). Activation of AMP-activated protein kinase (AMPK) signaling mediated NPC-26-induced CRC cell death. AMPKα1 shRNA knockdown or dominant negative mutation abolished NPC-26-induced AMPK activation and subsequent CRC cell death. NPC-26 disrupted mitochondrial function, causing mitochondrial permeability transition pore (mPTP) opening and reactive oxygen species (ROS) production. ROS scavengers (NAC or MnTBAP) and mPTP blockers (cyclosporin A or sanglifehrin A) blocked NPC-26-induced AMPK activation and attenuated CRC cell death. Significantly, intraperitoneal injection of NPC-26 potently inhibited HCT-116 tumor growth in severe combined immuno-deficient (SCID) mice. Yet, its anti-tumor activity was significantly weakened against AMPKα1-silenced HCT-116 tumors. Together, we conclude that NPC-26 kills CRC cells possibly via activating AMPK signaling.

## INTRODUCTION

Colorectal cancer (CRC) is still a major malignancy in the world, which causes significant mortality each year [1–3]. Mitochondrion is vital for regulating cell signaling and survival [4, 5]. Recent studies have developed a small-molecule mitochondrion-interfering compound, named NPC-26 [6]). It disturbs normal mitochondrial functions, causing mitochondrial permeability transition pore (mPTP) opening and reactive oxygen species (ROS) production, and eventually leading to cell death [6, 7]. NPC-26 induces a conversion from elongated to punctate mitochondria, and provokes non-apoptotic cell death which is BAX-/BAK-independent [6]. Further studies have proposed that NPC26-induced cell death is dependent on activation of kinase signaling pathways [6]. To our best knowledge, the potential effect of this compound in CRC cells has not been tested thus far. More importantly, the underlying signaling mechanisms of NPC-26-induced cell death are still vague. Here, we suggest that NPC-26 kills human CRC cells possibly via activating AMP-activated protein kinase (AMPK) signaling.

AMPK is the master energy sensor, which helps to maintain the balance of energy metabolism [8, 9]. Recent research has also proposed an important role of AMPK in mediating cell death (see review [10]). Cancer studies have demonstrated that multiple anti-cancer agents and natural occurring compounds could activate AMPK-dependent cell death pathways [10–21]. AMPK may induce cancer cell death via regulating multiple downstream signal targets, including in-activating cancer-promoting mammalian target of rapamycin (mTOR) signaling [22], phosphorylating pro-death p53 signaling [23] and provoking autophagy [24]. Disruption of mitochondrion was shown to provoke AMPK activation [25]. For instance, Head *et al*., demonstrated that itraconazole directly binds to and inhibits mitochondrial protein voltage-dependent anion channel 1 (VDAC1), causing ATP depletion and AMP:ATP ratio increase [25]. This would eventually lead to AMPK activation [25]. The current study suggests that AMPK activation also mediates NPC-26-induced killing of CRC cells.

## RESULTS

### NPC-26 is cytotoxic and anti-proliferative to cultured CRC cells

**Figure 1:**
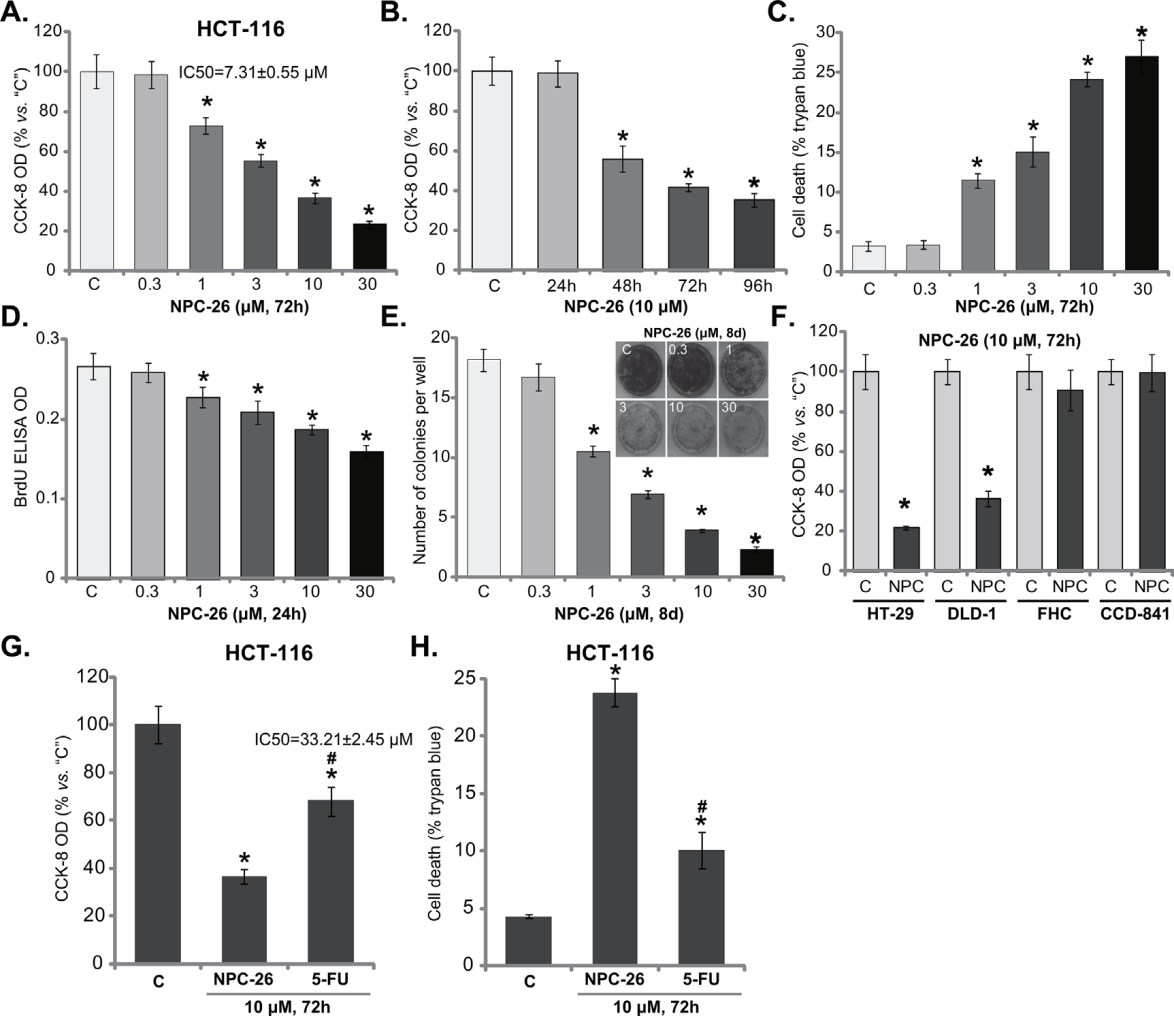
NPC-26 is cytotoxic and anti-proliferative to cultured CRC cells CRC cell lines (HCT-116, HT-29 and DLD-1) or normal colon epithelial cell lines (FHC and CCD-841) were either left untreated (“C”, same for all figures) or treated with designated concentration of NPC-26 (0.3-30 μM) or 5-Flurouracil (5-FU, 10 μM), cells were further cultivated in conditional medium for indicated time, cell survival **A**, **B**, **F** and **G**. cell death **C** and **H**. and proliferation **D** and **E**. were tested by listed assays. Data were expressed as mean ± SD (Same for all figures). For each assay, n=5. Experiments in this figure were repeated five times, and similar results were obtained each time. * ***p*** <0.05 *vs*. “C”. ^#^ ***p*** <0.05 *vs*. “NPC-26” (**G** and **H**).

First, the potential effect of NPC-26 on cultured CRC cells was tested. As demonstrated, treatment with NPC-26 (for 72 hours) in HCT-116 cells dose-dependently inhibited cell survival, which was tested by the CCK-8 OD reduction (Figure 1A). NPC-26, at over 1 μM, significantly decreased CCK-8 OD of HCT-116 cells (Figure 1A). NPC-26's IC-50, or the concentration that inhibited 50% of cell survival, was 7.31±0.55 μM (Figure 1A). NPC-26 also displayed a time-dependent response in inhibiting HCT-116 cell survival (Figure 1B). It would require 48 hours for 10 μM of NPC-26 to exert a significant effect (Figure 1B). When analyzing cell death, we showed that the number of trypan blue positive cells (“dead” cells) was significantly increased following 1-30 μM of NPC-26 treatment (Figure 1C). Thus, these results indicate that NPC-26 is cytotoxic to cultured HCT-116 cells.

We also tested the potential role of NPC-26 on CRC cell proliferation. BrdU ELISA assay was performed, and results showed that NPC-26 dose-dependently decreased the BrdU ELISA OD in HCT-116 cells (Figure 1D), indicating its anti-proliferative activity. Note that for the BrdU assay, cells were treated with NPC-26 for only 24 hours, when no significant cytotoxicity was yet noticed (Figure 1B). Further studies showed that NPC-26 at 1-30 μM dramatically decreased the number of proliferative HCT-116 colonies, again confirming its anti-proliferative activity (Figure 1E). Therefore, NPC-26 is also anti-proliferative to HCT-116 cells.

CCK-8 assay was again utilized to test the potential activity of NPC-26 to other CRC cells. Results showed that 10 μM of NPC-26 significantly inhibited survival of two other established CRC cell lines: HT-29 and DLD-1 (Figure 1F). On the other hand, same NPC-26 treatment (10 μM, 72 hours) failed to affect the survival of two normal colon epithelial cell lines: FHC and CCD-841 (Figure 1F). Thus, it appears that NPC-26 is only cytotoxic to the cancerous cells, which is proposed by other studies [7]. 5-Flurouracil (5-FU) in a very common chemotherapeutic drug for CRC treatment [26]. We showed that 10 μM of NPC-26 was more potent than same concentration of 5-FU in inhibiting HCT-116 cell survival (Figure 1G) and inducing cell death (Figure 1H). The IC-50 for 5-FU (72 hours treatment) was 33.21±2.45 μM (Figure 1G), which was also significantly higher than that of NPC-26 (7.31±0.55 μM).

### NPC-26-induced killing of CRC cells requires AMPK activation

**Figure 2:**
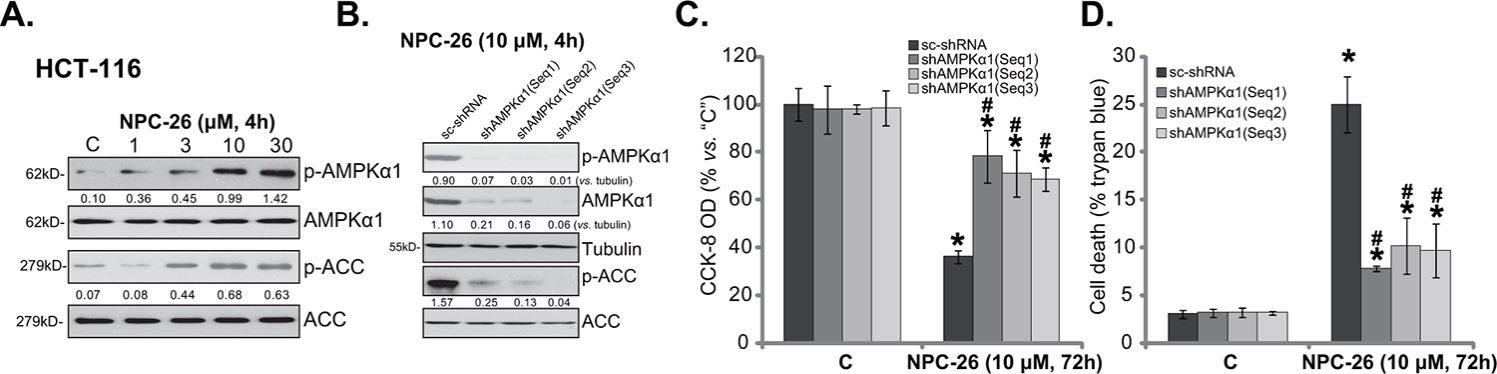
NPC-26-induced killing of CRC cells requires AMPK activation HCT-116 cells were treated with designated concentration of NPC-26 for 4 hours, expression of listed proteins was shown **A**. Puromycin-selected HCT-116 cells, expressing listed shRNA, were treated with/out NPC-26 (10 μM) for applied time, listed proteins were shown **B**.; Cell survival **C**. and cell death **D**. were tested. For each assay, n=5. Experiments in this figure were repeated three times, and similar results were obtained each time. AMPKα1/ACC phosphorylation (*vs*. total protein, or *vs*. tubulin when mentioned) was quantified (**A** and **B**). * ***p*** <0.05 *vs*. “C”. **^#^** ***p*** <0.05 *vs*. “sc-shRNA”.

As discussed, recent studies have shown that activation of AMPK by a number of different agents could kill CRC cells [13, 14, 27–29]. Thus, the possible involvement of AMPK signaling in NPC-26's actions was tested. Western blot assay results in Figure 2A demonstrated that treatment with NPC-26 (at 1-30 μM) in HCT-116 cells induced significant AMPK activation, the latter was tested by phosphorylation (“p-”) of AMPKα1 (T172) and its major downstream target acetyl-CoA carboxylase (ACC, Ser-79) [9]. NPC-26 again displayed a dose-dependent response in activating AMPK (Figure 2A, quantification).

Genetic strategy was then applied to interfere AMPK activation. First, three different AMPKα1 shRNAs with non-overlapping sequences [“shAMPKα1(Seq1/2/3)”] [30] were utilized to stably knockdown AMPKα1. As demonstrated, the three shRNAs indeed dramatically downregulated AMPKα1 in HCT-116 cells (Figure 2B). NPC-26-induced AMPK activation, or p-AMPKα1/p-ACC, was almost completely blocked in AMPKα1-silenced cells (Figure 2B). Consequently, NPC-26-induced cytotoxicity, tested by CCK-8 OD reduction (Figure 2C) and trypan blue staining increase (Figure 2D), was attenuated in AMPKα1-silenced HCT-116 cells. Notably, these AMPKα1 shRNAs alone didn't affect HCT-116 cell survival/death (Figure 2C and 2D).

### AMPKα1 mutation inhibits NPC-26-induced killing of HCT-116 cells

**Figure 3:**
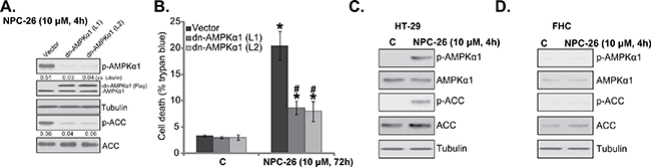
AMPKα1 mutation inhibits NPC-26-induced killing of HCT-116 cells Puromycin-selected HCT-116 cells, expressing dominant negative AMPKα1 (“dn-AMPKα1”, T172D) or empty vector, were treated with/out NPC-26 (10 μM) for applied time, listed proteins were shown **A**.; Cell death **B**. were tested. HT-29 cells or FHC colon epithelial cells were treated with designated concentration of NPC-26 for 4 hours, expression of listed proteins was shown **C**. and **D**. For each assay, n=5. Experiments in this figure were repeated three times, and similar results were obtained each time. AMPKα1/ACC phosphorylation (*vs*. total protein, or *vs*. tubulin when mentioned) was quantified (**A**). * ***p*** <0.05 *vs*. “C”. **^#^** ***p*** <0.05 *vs*. “Vector”.

The above shRNA results imply that AMPK activation mediates NPC-26-induced cytotoxicity against HCT-116 cells. To further support this hypothesis, a dominant negative AMPKα1 (“dn-AMPKα1”, T172D) construct [27, 31, 32] was introduced to the HCT-116 cells. Via puromycin selection, two stable HCT-116 cell lines with this construct were established [“dn-AMPKα1 (L1/2)”]. Western blot assay results in Figure 3A confirmed expression of dn-AMPKα1 (Flag-tagged) in the stable cells. Significantly, dn-AMPKα1 expression almost completely blocked NPC-26-induced AMPK activation (Figure 3A). As a result, NPC-26-induced HCT-116 cell death was also attenuated (Figure 3B). Interestingly, treatment with NPC-26 (10 μM, 4h) induced significant AMPK activation in HT-29 cells (Figure 3C), but not in the FHC colon epithelial cells (Figure 3D). As a matter of fact, expression of total AMPKα1 and ACC was also extremely low in the epithelial cells (Figure 3C and 3D). These results could at least in part explain why these epithelial cells were not killed by NPC-26 (Figure 1F). The above AMPKα1 shRNA and mutation experiments were also repeated in HT-29 cells, and similar results were obtained (Data not shown). Together, these results suggest that NPC-26-induced killing of CRC cells requires AMPK activation.

### NPC-26 disrupts mitochondrial function, causing AMPK activation

**Figure 4:**
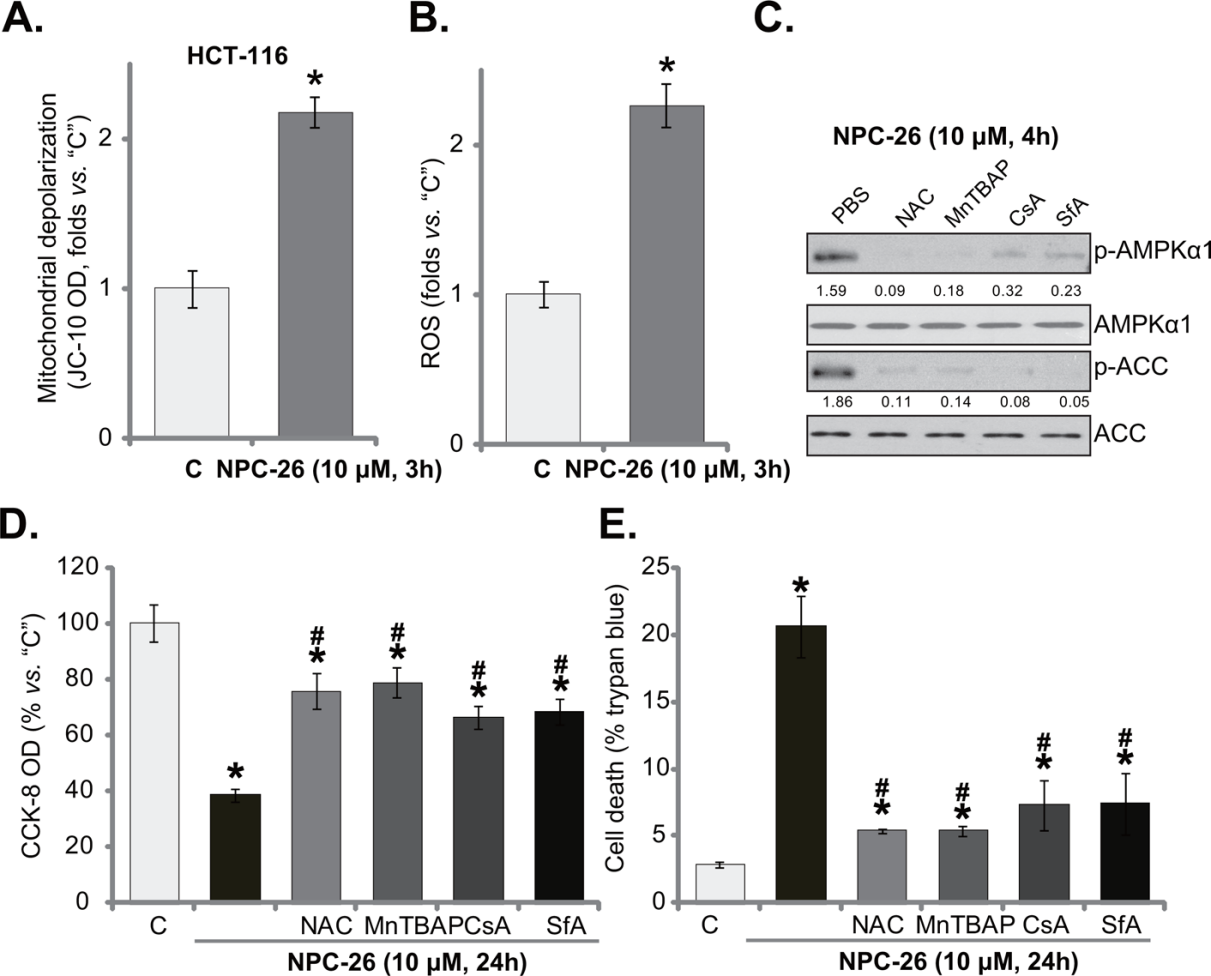
NPC-26 disrupts mitochondrial function, causing AMPK activation HCT-116 cells were treated NPC-26 (10 μM) for 3 hours, mitochondrial depolarization **A**. and cellar ROS content **B**. were tested. HCT-116 cells were pretreated for 30 min with NAC (500 μM), MnTBAP (10 μM), cyclosporin A (CsA, 0.5 μM), or sanglifehrin A (SfA, 2.5 μM), followed by NPC-26 (10 μM) treatment for indicated time period; Expression of listed proteins were shown **C**.; Cell survival **D**. and cell death **E**. were also tested. For each assay, n=5. Experiments in this figure were repeated three times, and similar results were obtained each time. AMPKα1/ACC phosphorylation (*vs*. total protein) was quantified (**C**). * ***p*** <0.05 *vs*. “C”. **^#^** ***p*** <0.05 *vs*. NPC-26 only.

A very recent study by Dong *et al*., [7] demonstrated that NPC-26 disrupts normal mitochondrial function, leading to mPTP opening and ROS production in pancreatic cancer cells. Here, NPC-26 treatment (10 μM) in HCT-116 cells similarly induced mitochondrial depolarization (JC-10 intensity increase, indicating mPTP opening [7]) (Figure 4A) and significant ROS production (Figure 4B). Thus, normal mitochondrial functions could also be disrupted by NPC-26 in HCT-116 cells. To study the link between mitochondrial dysfunction and AMPK activation in NPC-26-treated cells, pharmacological strategy was applied. As demonstrated in Figure 4C, NPC-26-induced AMPK activation was largely inhibited with co-treatment of ROS scavengers (NAC and MnTBAP [33]) or mPTP blocker cyclosporin A (CsA) [34] and sanglifehrin A (SfA) [35]. Remarkably, above inhibitors also alleviated NPC-26-induced killing of HCT-116 cells (Figure 4D and 4E). Treatment with these inhibitors alone didn't affect AMPK activation and HCT-116 cell survival/death (Data not shown). These results suggest that NPC-26 induces mitochondrial dysfunction, which possibly leads to AMPK activation and subsequent cell death.

### NPC-26 inhibits HCT-116 tumor growth in SCID mice

**Figure 5:**
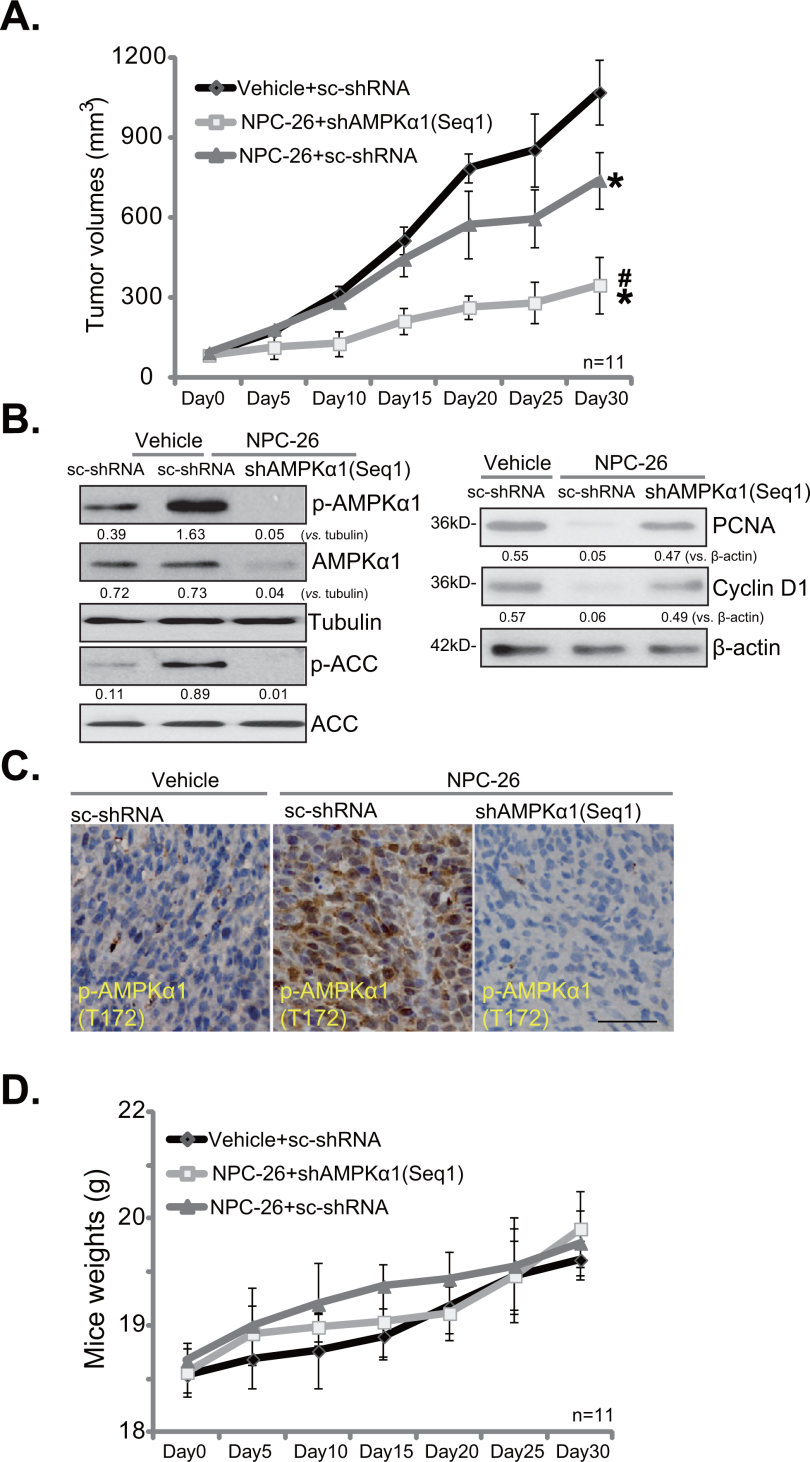
NPC-26 inhibits HCT-116 tumor growth in SCID mice Same amount (5 million per mouse) of HCT-116 cells expressing scramble control shRNA (“sc-shRNA”) or AMPKα1 shRNA (“Seq1”) were inoculated to the SCID mice to establish xenografted tumors. Mice were administrated with NPC-26 (25 mg/kg, daily, *i.p*.) or saline (“Vehicle”) for a total of 30 days, tumor volume **A**. and mice body weight **D**. were recorded every 5 days. Twelve hours after initial NPC-26 administration, one tumor of each group was isolated, and expression of listed proteins was tested by Western blot assay **B**. or IHC staining assay **C**. * ***p*** <0.05 *vs*. “Vehicle”. **^#^** ***p*** <0.05 *vs*. NPC-26 treatment of “sc-shRNA” tumors. Bar=100 μm (C).

In order to test the anti-tumor activity of NPC-26 *in vivo*, the severe combined immuno-deficient (SCID) mice bearing HCT-116 xenograft tumor model was applied. Results in Figure 5A demonstrated that *i.p*. injection of NPC-26 (25 mg/kg body weight, daily) [7] dramatically suppressed growth of HCT-116 tumors with scramble control shRNA (“sc-shRNA”, or control tumors). Significantly, NPC-26-induced anti-tumor activity *in vivo* was remarkably weakened against HCT-116 tumors with AMPKα1 shRNA (“Seq-1”) (Figure 5A), indicating that AMPK activation could also be required for NPC-26's actions *in vivo*. Indeed, when analyzing tumor tissue samples, we found that NPC-26 administration (12 hours after initial administration) induced significant AMPK activation, or p-AMPK/p-ACC, in control tumors (Figure 5B, left panel), which was absent in tumors expressing AMPKα1 shRNA (Figure 5B, left panel). Tumors with AMPKα1 shRNA also showed extremely low expression of total AMPKα1 (Figure 5B, left panel). Proliferating cell nuclear antigen (PCNA) expression is a well-established marker of proliferation. Cyclin D1 is important for cell proliferation [36, 37]. Here, we showed that PCNA and Cyclin D1 were both downregulated in NPC-26-treated control tumors, but not in AMPKα1-silenced tumors (Figure 5B, right panel). IHC staining assay further confirmed AMPK activation [p-AMPKα1 (Thr-172) staining] by NPC-26 in control tumors, but not in the AMPKα1 shRNA-expressing tumors (Figure 5C). Notably, expression of AMPKα1 shRNA alone didn't affect HCT-116 tumor growth in SCID mice. Results in Figure 5D demonstrated that the above NPC-26 administration didn't affect the body weight of experimental mice, indicating that the regimens were relatively safe [7].

## DISCUSSION

AMPK plays a pivotal role in regulating a number of key cellular functions, from energy metabolism, cell mitosis, apoptosis to autophagy [9, 38]. However, whether AMPK is pro-survival or pro-death is still debatable. It is now known that sustained or intensified AMPK activation will inhibit cell growth and promote cancer cell death [13, 39, 40]. As a matter of fact, a number of anti-cancer agents were shown to kill cancer cells via activating AMPK-dependent signalings [10–21].

Under certain circumstances, it has yet been proposed that AMPK activation could also be pro-survival [41, 42]. The difference might be due to the intensity of AMPK activation. Low level of AMPK activation might promote cell survival, but intensified AMPK activation could promote cell death via regulating its downstream signalings (p53, mTOR inhibition and autophagy *etc*.). In fact, the activity of AMPK could increase over 100-fold on phosphorylation of a conserved threonine residue (Thr-172) within the activation loop at α1 subunit [38, 43]. In the current study, we showed that NPC-26 induced significant AMPKα1 phosphorylation at Thr-172, indicating a profound AMPK activation. Remarkably, AMPKα1 shRNA knockdown or Thr-172 dominant negative mutation not only abolished NPC-26-induced AMPK activation, but also attenuated CRC cell death. Thus, AMPK activation by NPC-26 is indeed pro-death in CRC cells. Notably, NPC-26 was non-cytotoxic to normal colon epithelial cells, where AMPK was also not significantly provoked. Further studies showed that NPC-26 disrupted mitochondrial function, causing mPTP opening and ROS production, which served as the upstream signal for AMPK activation. Remarkably, ROS scavengers (NAC or MnTBAP) and mPTP blockers (CsA or SfA) almost completely blocked NPC-26-induced AMPK activation.

It should be noted that mitochondria in cancer cells are structurally and functionally different from those in normal (“epithelial”) cells, which are often highly-active in malignant cells to participate in metabolic reprogramming and cell activities [4, 5]. Intriguingly, existing literatures have also reported that certain key mPTP components are up-regulated in cancer cells. For example, the ATP synthase c subunit was upregulated in human breast cancer cells [44]. VDAC-1 over-expression was also observed in several cancer cells [7, 45]. Unique upregulation of mPTP components and high mitochondrial activity in cancer cells could explain why only cancer cells, but not the epithelial cells, were killed by NPC-26 treatment. As a matter of fact, we found that NPC-26 failed to induce ROS production, AMPK activation and significant cytotoxicity in two normal colon epithelial cell lines (FHC and CCD-841). The selective cytotoxicity of NPC-26 to cancerous cells has been reported early as well [7].

Intriguingly, AMPK blockage, via AMPKα1 shRNA knockdown or Thr-172 dominant negative mutation, didn't completely abolished NPC-26-mediated killing of CRC cells (Figure 2 and 3). Meanwhile, ROS scavengers (NAC or MnTBAP) as well as mPTP blockers (CsA and SfA) only alleviated, but didn't abolish NPC-26's cytotoxicity (Figure 4). It is possible that these interfering strategies didn't result in complete inhibition of the targeted pathways (AMPK, ROS and mPTP). It is more likely that other signalings besides AMPK may also contribute to NPC-26's actions in CRC cells. Therefore, further studies will be needed to explore the relationship between AMPK and these other pathways in mediating NPC-26's actions in CRC cells. It will also be important to further characterize the underlying mechanism of NPC-26-induced AMPK activation. In summary, we propose that NPC-26 kills CRC cells possibly via activating AMPK signaling. NPC-26 might have translational value for the treatment of CRC.

## MATERIALS AND METHODS

### Chemicals, reagents, and antibodies

NPC-26 was a gift from Dr. Dong's group [33]. N-acetyl cysteine (NAC) and Mn (III) tetrakis (4-benzoic acid) porphyrin (MnTBAP), a superoxide dismutase mimetic, were provided by Sigma Chemicals (Sigma, St. Louis, MO). mPTP blockers cyclosporin A (CsA) or sanglifehrin A (SfA) and 5-Flurouracil (5-FU) were also obtained from Sigma Chemicals. Antibodies for tubulin and AMPK signaling proteins were purchased from Cell Signaling Tech (Shanghai, China).

### Cell culture

HCT-116, DLD-1 and HT-29 CRC cell lines were provided by Dr. Lu's group [27, 46]. Cells were maintained in DMEM medium (with 10% FBS). Two normal colon epithelial cell lines, FHC and CCD-841, were purchased from the Cell Bank of Shanghai Institute of Biological Science (Shanghai, China). The above epithelial cells were also cultivated in DMEM medium.

### Cell survival assay

To test cell survival, cell counting kit-8 (CCK-8, Dojindo Laboratories, Kumamoto, Japan) assay kit was performed based on the attached manual. CCK-8 absorbance optic density (OD) was recorded at 450 nm.

### Trypan blue staining assay

After treatment, trypan blue (0.2%)-stained cells was counted by the Countess automatic cell counter (Invitrogen, Shanghai, China). The cell death percentage (%) was calculated by the number of the trypan blue cells divided by the total cell number.

### BrdU incorporation assay

To test cell proliferation, 5-bromo-2’-deoxyuridine (BrdU) incorporation assay was applied. Briefly, BrdU (10 μM, Roche, Shanghai, China) was pre-added. Following treatment of cells, BrdU incorporation was determined in enzyme-linked immunosorbent assay (ELISA) format. ELISA OD at 450 nm was tested.

### Colony formation assay

HCT-116 cells with applied NPC-26 treatment were re-suspended in DMEM medium containing 0.5% agar (Sigma), which were then plated onto a pre-solidified six-well plate. Afterwards, cells were cultured in NPC-26-containing medium for 8 days. The remaining colonies were counted manually.

### Western blot assay

Cell or HCT-116 tumor tissue samples were incubated in lysis buffer described [27, 46]. Quantified protein lysates (30 μg/lane) were separated by 8-10% of SDS-PAGE gels, and were transferred onto polyvinylidene difluoride (PVDF) membranes. The membranes were then blocked with 10% milk, and incubated with designated primary and secondary antibodies. The blots were then subjected to ECL detection. Indicated protein band was quantified of total gray via ImageJ software (NIH).

### Mitochondrial depolarization assay

As described previously [7], mitochondrial depolarization, indicating mPTP opening, was tested by the commercial JC-10 dye (Invitrogen) [7]. Following the applied treatment, CRC cells were incubated with JC-10 dye (2 μg/mL). Afterwards, the green fluorescence intensity was tested immediately via a fluorescence microplate reader (Titertek Fluoroscan, Germany) [7].

### ROS detection

Following treatment, cells were stained with CellRox Orange Reagent (5 μM, Invitrogen) at 37°C for 30 min. ROS content was detected by the above fluorescence microplate reader (Titertek Fluoroscan, Germany).

### AMPKα1 short hairpin RNA (shRNA) knockdown

Three AMPKα1 shRNAs [31] with non-overlapping sequences (“Seq-1/2/3”) were designated by Genepharm (Shanghai, China). The short hairpin sequences for AMPKα1 were : 5’-GCAGAAGTTT GTAGGGCAATT-3’(shAMPKα1-Seq1) [47], 5’-GCATAA TAAGTCACAGCCAAA-3’ (shAMPKα1-Seq2) [48] and 5’CTCCAAGACCAGGAAGTCATACAATAGAA-3’(shAMPKα1-Seq3) [49]. The AMPKα1 shRNAs were packed into GV248 lentiviral vector containing puromycin resistance gene. The lentiviral AMPKα1 shRNA was added to HCT-116 cells for 12 hours. Afterwards, puromycin (5 μg/mL) was added to select stable colonies for 5-6 passages. AMPKα1 expression in the stable cells was determined by Western blot assay. Control cells were infected with same amount of lentiviral scramble shRNA.

### AMPK dominant negative mutation

Dominant-negative (dn) mutant of AMPKα1 (AMPKα1-T172A) construct was provided by Dr. Lu's group [27, 31, 32]. The dn-AMPKα1 or the empty vector (each 0.2 μg/mL per well) was transfected to cancer cells via Lipofectamine 2000, and stable cells were again selected by puromycin (5 μg/mL).

### Mice xenograft assay

The protocols using 6-8 week-old severe combined immunodeficient (SCID) mice (weighting 18-19g) were approved by the IACUC of all authors’ institutions. Exponentially growing HCT116 cells (5*10^6^ cells per mouse), expressing scramble control shRNA or AMPKα1 shRNA (“Seq1”), were subcutaneously (*s.c*.) injected into SCID mice. Within three weeks, the xenografted tumors were established around 100 mm^3^ in volume. The SCID mice were then treated as described. Tumor volumes, recorded every 5 days, were calculated using the formula: (mm^3^) = (A^2^ × B)/2: A and B were the shortest and the longest diameter, respectively. Mice body weights were also recorded.

### Immunohistochemistry (IHC) staining

The staining was performed on the cryostat sections (4 μm) of HCT-116 tumors using the standard protocol [32]. p-AMPKα1 (Thr-172, 1: 50) antibody was utilized. DAB was applied to stain the positive staining.

### Statistical analysis

All values were expressed as the mean ± standard deviation (SD). A *p*-value, calculated by ANOVA, of less than 0.05 was considered statistically significant.
